# Assessing the sustainability capacity of evidence-based programs in community and health settings

**DOI:** 10.3389/frhs.2022.1004167

**Published:** 2022-11-30

**Authors:** Caren Bacon, Sara Malone, Kim Prewitt, Rachel Hackett, Molly Hastings, Sarah Dexter, Douglas A. Luke

**Affiliations:** ^1^Center for Public Health Systems Science, Brown School, Washington University in St. Louis, St. Louis, MO, United States; ^2^Division of Public Health Sciences, Department of Surgery, Washington University in St. Louis School of Medicine, St. Louis, MO, United States

**Keywords:** sustainability capacity, implementation science, program sustainability, evidence-based interventions, community, health

## Abstract

**Background:**

Within many public health settings, there remain large challenges to sustaining evidence-based practices. The Program Sustainability Assessment Tool has been developed and validated to measure sustainability capacity of public health, social service, and educational programs. This paper describes how this tool was utilized between January 2014 and January 2019. We describe characteristics of programs that are associated with increased capacity for sustainability and ultimately describe the utility of the PSAT in sustainability research and practice.

**Methods:**

The PSAT is comprised of 8 subscales, measuring sustainability capacity in eight distinct conceptual domains. Each subscale is made up of five items, all assessed on a 7-point Likert scale. Data were obtained from persons who used the PSAT on the online website (https://sustaintool.org/), from 2014 to 2019. In addition to the PSAT scale, participants were asked about four program-level characteristics. The resulting dataset includes 5,706 individual assessments reporting on 2,892 programs.

**Results:**

The mean overall PSAT score was 4.73, with the lowest and highest scoring subscales being funding stability and program adaptation, respectively. Internal consistency for each subscale was excellent (average Cronbach's alpha = 0.90, ranging from 0.85 to 0.94). Confirmatory factor analysis highlighted good to excellent fit of the PSAT measurement model (eight distinct conceptual domains) to the observed data, with a comparative fit index of 0.902, root mean square error of approximation equal to 0.054, and standardized root mean square residual of 0.054. Overall sustainability capacity was significantly related to program size (*F* = 25.6; *p* < 0.001). Specifically, smaller programs (with staff sizes of ten or below) consistently reported lower program sustainability capacity. Capacity was not associated with program age and did not vary significantly by program level.

**Discussion:**

The PSAT maintained its excellent reliability when tested with a large and diverse sample over time. Initial criterion validity was explored through the assessment of program characteristics, including program type and program size. The data collected reinforces the ability of the PSAT to assess sustainability capacity for a wide variety of public health and social programs.

## Introduction

What enables programs to continue delivering effective services over time? This is an important question funders and public health leaders pose as they look beyond the initial investment and implementation of a program. As Shelton et al. (1) note, funders want to know the investment they make into a program will continue to have an impact long after the investment ends. In addition, communities come to rely on these programs and if they end prematurely it may have lasting consequences. The discontinuation of these programs results in communities developing “low levels of community support and trust in research and public health/medical institutions” therefore creating challenges to future community efforts (1, 2). Program sustainability has been identified as critical for realizing the long-term impacts of a program. However, researchers and practitioners still lack knowledge about how to measure or enhance sustainment of public health or clinical care programs. The growing evidence base in dissemination and implementation science focuses mostly on the translation of research into practice to develop effective programs and policies (1). Many implementation science studies focus on the early stages of implementation and short-term outcomes; thus there is an important research and evaluation opportunity focusing on how effective programs are sustained over time after their initial adoption (1, 3, 4).

Sustainability has been defined as the ongoing use of an intervention with enough fidelity to continue to have desired program impact with subsequent improved outcomes (1). In addition to this foundational definition, recent empirical work has examined various research and measurement aspects of assessing sustainment in public health settings (5–8). Together, this information to improve sustained implementation will help both researchers and practitioners realize the full impact of their programs and practices.

Despite these research successes, it remains challenging for practitioners to maintain evidence-based activities and programs across a wide range of settings. Public health and community programs often depend on time-limited financial resources, after which programs are expected to secure alternative funding (9). Programs may also lose political and community support, become targets of political or commercial opposition, or face organizational challenges such as staff turnover (1). Sustaining programs that work is the main way we can ensure that communities get their intended health benefits; for that reason it is critical to be able to measure and understand the factors influencing program sustainability.

Public health program sustainability can take many forms (10). For example, practitioners can seek to maintain program activities, community-level partnerships, organizational practices, benefits to clients, and the salience of the program's core issue (11). However, little is known about how a program can best position itself to deliver these outcomes over time. Research and theory on the concept of value-based care has also focused on some of these organizational activities and describes a need to focus on overall value of care and team-based care instead of simply focusing on reducing costs of care (12). By focusing on building sustainability capacity, or the structures and processes that allow a program to leverage resources to effectively implement and maintain evidence-based policies and activities, programs can better understand and strengthen the factors within their control to increase the likelihood of maintaining benefits to clients in some form over time (10).

**Figure 1:**
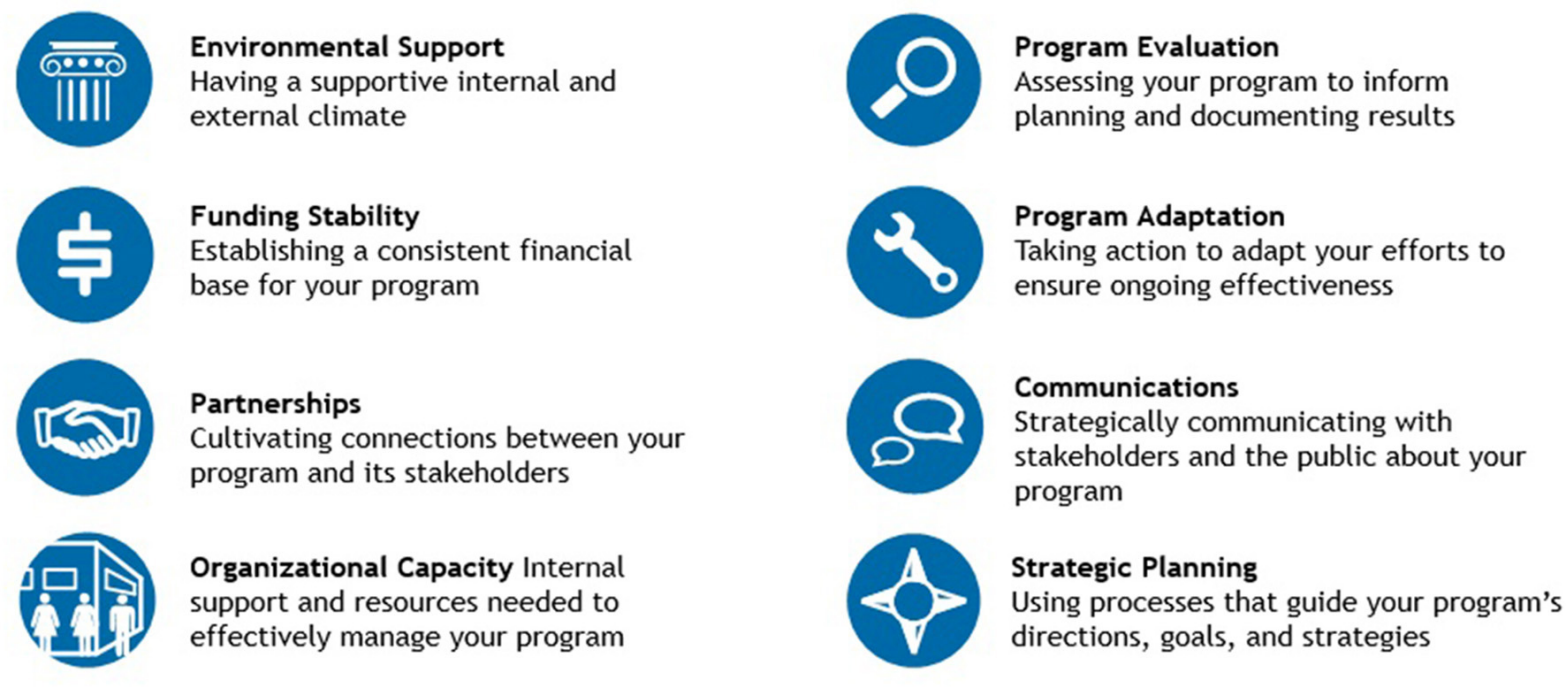
Conceptual domains for the program sustainability framework.

To better understand the factors that affect a program's ability to deliver benefits over time, Schell et al. (10) developed a sustainability conceptual framework using concept mapping, reviews of the implementation science literature, and expert input. This framework identifies a set of organizational factors affecting program sustainability capacity. These factors are organized into external (environmental support, funding stability) and internal (partnerships, organizational capacity, program evaluation, program adaptation, communications, and strategic planning) domains (see Figure 1).

This eight-domain conceptual framework is useful to help programs and organizations looking to understand the factors beyond simple funding that affect a program's capacity for sustainability. For example, programs that collect evaluation data about their processes and outcomes can better demonstrate the necessity of their program to leadership and stakeholders (13). Communicating concise outcome data to policymakers and showing a program's impact on the community better positions the program for continued funding and support (14).

To address the relative lack of tools available to evaluate program sustainability, Luke et al. (15) translated the program sustainability conceptual framework into a measurement instrument: the Program Sustainability Assessment Tool (PSAT). The development of the tool was guided by four basic design principles: (1) short and easy to use; (2) usable by both small and large programs; (3) applicable to a wide variety of program types; and (4) useful as a research, evaluation, and program planning tool.

The PSAT was originally created to reflect the concepts represented in each of the eight domains in the program sustainability framework (10). The assessment instrument was developed and tested on over 250 state and local public health programs across a variety of program types (tobacco control, obesity, nutrition, etc.). Psychometric analyses of the PSAT using these original data demonstrated good reliability (i.e., internal consistency) and confirmatory factor analysis supported the suitability of eight domains measured by five items per domain (15).

The PSAT was designed to allow comparisons between programs as well as within-program comparisons over time. Since 2014, the tool has been used by more than 5,000 people to rate sustainability capacity of more than 2,500 public health, social services, clinical care, and education programs of varying sizes in the US and internationally. Among many others, the PSAT has been used to examine the sustainability capacity of inter-professional collaborative practice model for population health, evidence-based practices in adolescent substance abuse, local health department programs and policies, and chronic disease prevention interventions (16–21).

The variety of settings and amount of use provides the opportunity for further exploration of program sustainability capacity across program type, size, implementation level, and age. An example application of the PSAT in public health programming is how the Centers for Disease Control & Prevention (CDC)'s Office on Smoking and Health requires state tobacco control programs create a sustainability plan and encourages programs to use the Program Sustainability Assessment Tool to drive sustainability planning (22). Additional departments within CDC (Division of Population Health, Division of Nutrition, Physical Activity and Obesity, and the Center for Putting Prevention to Work, among others) have encouraged their state programs to use the PSAT, as have other federal organizations (National Cancer Institute, Canadian Partnership Against Cancer), and many state health departments, health foundations, and professional networks (American Evaluation Association, Association of State Public Health Nutritionists, and CDC-OSH National Partner Network) (23).

In this paper we describe program sustainability capacity for public health, clinical care, social services, and education programs using data passively collected on https://sustaintool.org between January 2014 and January 2019. We also examine how a small set of programmatic factors are related to program sustainability capacity. This paper adds to the sustainability knowledge base in two important ways, first, by identifying characteristics of programs that are associated with organizational capacity for sustainability, and second by providing further evidence supporting the utility of the PSAT as a continuing part of the implementation science sustainability toolkit.

## Methods

In this paper we report updated reliability, measurement model characteristics, and validity data for the Program Sustainability Assessment Tool (PSAT), which can be used to assess capacity for sustainability of a wide variety of public health, social service, and educational programs.

### Measures

The PSAT (Additional File 1) consists of 40 questions, five items in each of eight domain subscales, with 7-point Likert-scale responses. Individual items can be rated from 1 (program has or does this to *no* extent) to 7 (program has or does this to the *full* extent). Subscale and total scores are the averages of the individual item scores, so scores can range from 1 to 7. Higher scores are interpreted as the program having greater sustainability capacity in that area (e.g., funding stability, program evaluation, etc.).

In addition to the PSAT total scale and subscale scores, participants provided information on four important program-level characteristics. *Program type* classified programs into five groups: public health, social service, clinical care, education, and other. As an example of a clinical setting, the PSAT has been used to examine the sustainability capacity of pediatric asthma care coordination (17). Public health programs both in the United States and abroad used the assessment. The Reducing Violence against Women and their Children grants program used the PSAT for funded prevention initiatives in diverse settings across Victoria, Australia to prevent violence against women (24). In the United states, Well-Ahead Louisiana, the state tobacco cessation and prevention program used the PSAT to assess their comprehensive statewide tobacco prevention efforts (25).

*Program level* captured how the program was organized and who it served. Programs were either community-level, state-level, or greater than state level. This latter category included national, tribal, and international programs. An example of a community-level program is the “Som la Pera” intervention; a school-based, peer-led, social-marketing intervention that encourages healthy diet and physical activity, in low socioeconomic adolescents (26). *Staff size* was the number of staff and personnel who were directly involved with the program or project being rated, including volunteers. *Program age* was the number of years that the program or project had been in existence.

### PSAT data collection

The PSAT analyses presented here are based on PSAT profile and data passively collected on https://sustaintool.org/ between January 2014 and January 2019 through individual and group self-assessments. Per the site privacy statement (with associated Washington University IRB approval), users passively consented to analysis of their de-identified PSAT profile data upon submission.

After downloading the PSAT data from the web server, the raw data were cleaned up by deleting test entries, and entries that had missing data for every item in the PSAT. (These were due to people who visited the website, started the PSAT assessment, but quit before filling anything out.) After cleaning, the dataset included a total of 5,706 respondents reporting on 2,892 programs. Examining missing data patterns, 65% of the respondents filled out every one of the 40 items in the scale, and 96% filled out at least half of the items (≥20).

Users of the online PSAT can fill out an individual assessment (one person rating the sustainability capacity of an individual program), or a group assessment (multiple people rating the sustainability capacity of the same program). Of the 2,892 program assessments included in the dataset, 2,283 were individual assessments (79%). For group assessments, the respondent numbers ranged from 2 to 31, with a median group size of 5. The main purpose of this paper is to understand characteristics of program sustainability capacity, so the raw data were aggregated by program. Specifically, the group PSAT total and subscale scores were calculated by averaging the scores for all individuals taking part in a particular group assessment. So, the scores are meant to represent program and organizational characteristics, not individual characteristics.

Table 1 presents the program-level characteristics of the total PSAT sample. This sample includes a wide variety of types of programs. Almost half of the programs are public health (46%), followed by social service (20%), clinical care (15%), and education (10%). A large majority of the programs are organized at the community level (74%), but over 700 programs are organized at higher levels (e.g., state, national, international). Programs represent both small and larger organizations, ranging from 3 or fewer staff members (21%) to more than 20 members (25%). The programs also varied in age, ranging from < 1 year of existence (26%) to over 3 years (45%).

**Table 1:** Program characteristics of PSAT sample (*N* = 2,892 programs, based on 5,706 individual assessments).

**Program characteristic**	**Number**	**%**
**Program type**
Public health	1,322	45.7
Social services	585	20.2
Clinical care	425	14.7
Education	293	10.1
Other	267	9.2
**Program level**
Community	2,082	73.8
State	452	16.0
Beyond state	285	10.1
**Staff size**
1–3	566	21.2
4–10	988	36.9
11–20	453	16.9
>20	668	25.0
**Program age**
< 1 year	696	25.5
1–3 years	804	29.5
>3 years	1,228	45.0

### Analyses

Frequencies and means were calculated to obtain descriptive statistics of the sample, as appropriate. One-way analyses of variance were conducted to understand PSAT score differences related to program focus, size, age and level. Two-way analyses of variance were conducted to assess the interaction between program focus and program size, age and level. Psychometric analyses were conducted to assess the reliability (internal consistency) of the eight PSAT subscales. Finally, confirmatory factor analysis was used to test the measurement model of the PSAT, and how well that measurement model fit with the observed PSAT data.

## Results

The goal of these analyses is to describe the characteristics of the Program Sustainability Assessment Tool (PSAT) as it has been applied to rate sustainability capacity in a variety of settings and programs. These analyses can help determine if the psychometric properties have remained stable as the PSAT has been rolled out for wider application, and to assess how a small number of program characteristics are related to PSAT overall and subscale scores.

### Overall PSAT characteristics

**Figure 2:**
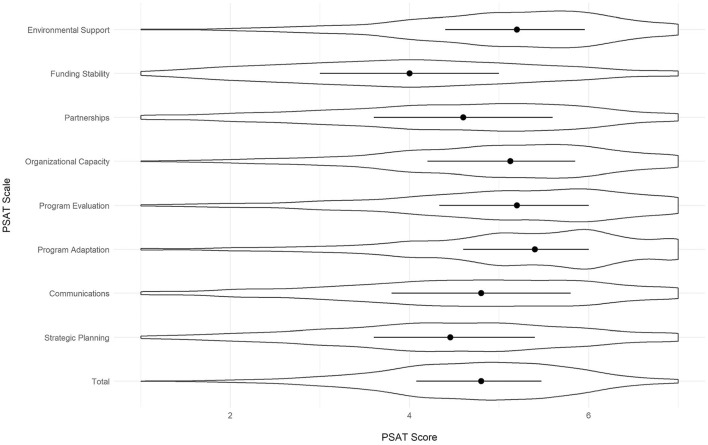
Variability of PSAT total and subscale scores (*N* = 2,892 programs).

All of the scores for the PSAT were out of a possible total of 7, with 7 being the greatest extent of each domain. Across all programs, the mean PSAT score was 4.73 (Table 2). PSAT subscale scores were lowest for funding stability (*M* = 3.97), followed by strategic planning (*M* = 4.42), partnerships (*M* = 4.47), communications (*M* = 4.64), organizational capacity (*M* = 4.98), program evaluation (*M* = 5.08), environmental support (*M* = 5.08) and program adaptation had the highest average score (*M* = 5.23). Although the subscale and total mean scores are somewhat high relative to the seven-point scale, score variabilities are relatively high (standard deviations ranging from 1.13 to 1.43), indicating only minor issues with restriction of range. Figure 2 presents violin plots of the total and subscale scores, displaying the median values for each, as well as the score variabilities.

**Table 2:** PSAT subscale characteristics and reliabilities.

**Scale**	**Mean**	**SD**	**Cronbach's α**
Environmental support	5.08	1.13	0.85
Funding stability	3.97	1.41	0.88
Partnerships	4.47	1.43	0.92
Organizational capacity	4.98	1.22	0.89
Program evaluation	5.08	1.31	0.91
Program adaptation	5.23	1.23	0.92
Communications	4.64	1.42	0.94
Strategic planning	4.42	1.36	0.89
Total PSAT scale	4.73	1.04	NA

### PSAT reliabilities and measurement structure

In our original PSAT development study, average internal consistency (Cronbach's alpha) of the 8 subscales was 0.88 and domain subscales ranged from 0.79 to 0.92 (15). In the current study, we had data on more programs, and these programs were more diverse (i.e., educational, clinical, social service, and public health programs). Despite the greater program diversity, psychometric analyses reveal that the PSAT maintains its excellent reliability (Table 2). Specifically, for the new data subscale reliabilities ranged from 0.85 to 0.94, with an average of 0.90.

In addition to the subscale reliabilities, we examined the domain structure of the PSAT using confirmatory factor analysis (CFA) to see how well the observed data matched our overall conceptual framework of eight distinct conceptual domains (see Figure 1). CFA results show an excellent fit of the data to the hypothesized measurement structure. Specifically, the fit indices for the eight factor model include the comparative fit index (CFI = 0.902), root mean square error of approximation (RMSEA = 0.054) and standardized root mean square residual (SRMR = 0.054). All indicate good to excellent fit (27–29). Furthermore, we compared the fit of the eight factor model to a simpler single factor model (that assumes that there is just a general concept of sustainability capacity that does not have a more complicated multi-domain structure). A comparison of the two models using Vuong's distinguishability test showed that the eight factor model was a significantly better fit to the data than the single factor model (LR = 47,277.2, *p* < 0.001) (30). More detailed results from the CFA analyses (including model fits and diagnostics) are available from the authors.

### PSAT characteristics by program type

**Figure 3:**
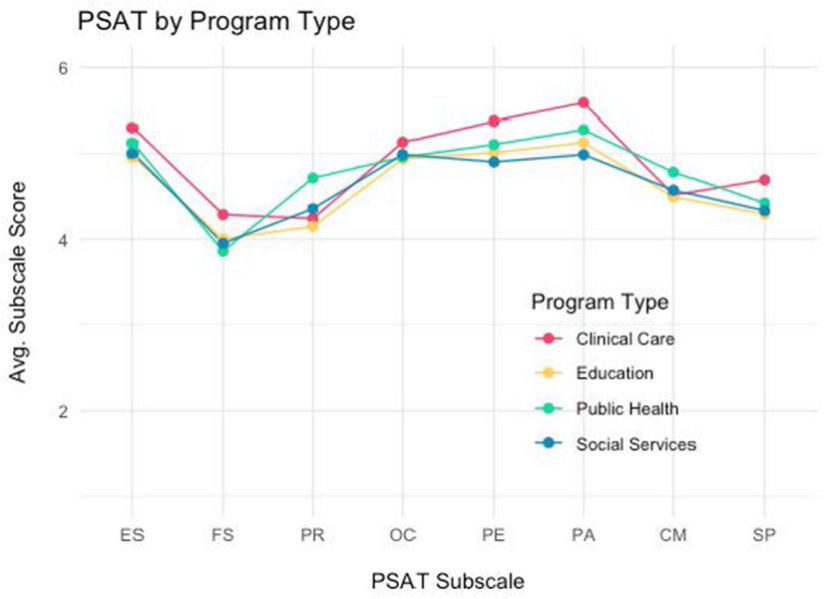
PSAT subscale scores by type of program.

PSAT scores were analyzed by program type for four types of programs: public health, social services, clinical care and education. Overall, PSAT total and subscale scores varied significantly by program type, except for organizational capacity (see Table 3). Clinical programs reported the highest total PSAT scores (*M* = 4.92), followed by public health (*M* = 4.78), social services (*M* = 4.64), and finally education (*M* = 4.61). Figure 3 shows the pattern of PSAT total and subscale scores by the four types of programs. Clinical programs tended to show higher subscale scores, especially for engaged stakeholders, financial stability, program evaluation, and program adaptation. Public health programs, on the other hand, showed higher scores on partnerships and communications. Social service programs have the lowest score profile of the four program types, with the possible exception of partnerships.

**Table 3:** Tests of PSAT subscales score differences by program type, level, staff size, and age.

**Scale**	**Program type**		**Program level**		**Staff size**		**Program age**	
	***F***	***p***	***F***	***p***	***F***	***p***	***F***	***p***
Environmental support	7.92	0.000	0.10	0.903	21.08	0.000	0.26	0.773
Funding stability	10.10	0.000	3.06	0.047	17.25	0.000	7.03	0.001
Partnerships	22.91	0.000	1.03	0.358	27.22	0.000	1.19	0.305
Organizational capacity	2.38	0.068	2.39	0.092	20.06	0.000	0.24	0.791
Program evaluation	11.20	0.000	0.37	0.692	16.34	0.000	8.54	0.000
Program adaptation	21.11	0.000	2.19	0.112	5.54	0.001	7.26	0.001
Communications	6.82	0.000	6.95	0.001	9.11	0.000	2.68	0.069
Strategic planning	7.34	0.000	2.14	0.117	16.23	0.000	3.04	0.048
Total PSAT scale	8.66	0.000	0.61	0.542	25.56	0.000	1.49	0.226

### Impact of programmatic factors on PSAT trends

**Figure 4:**
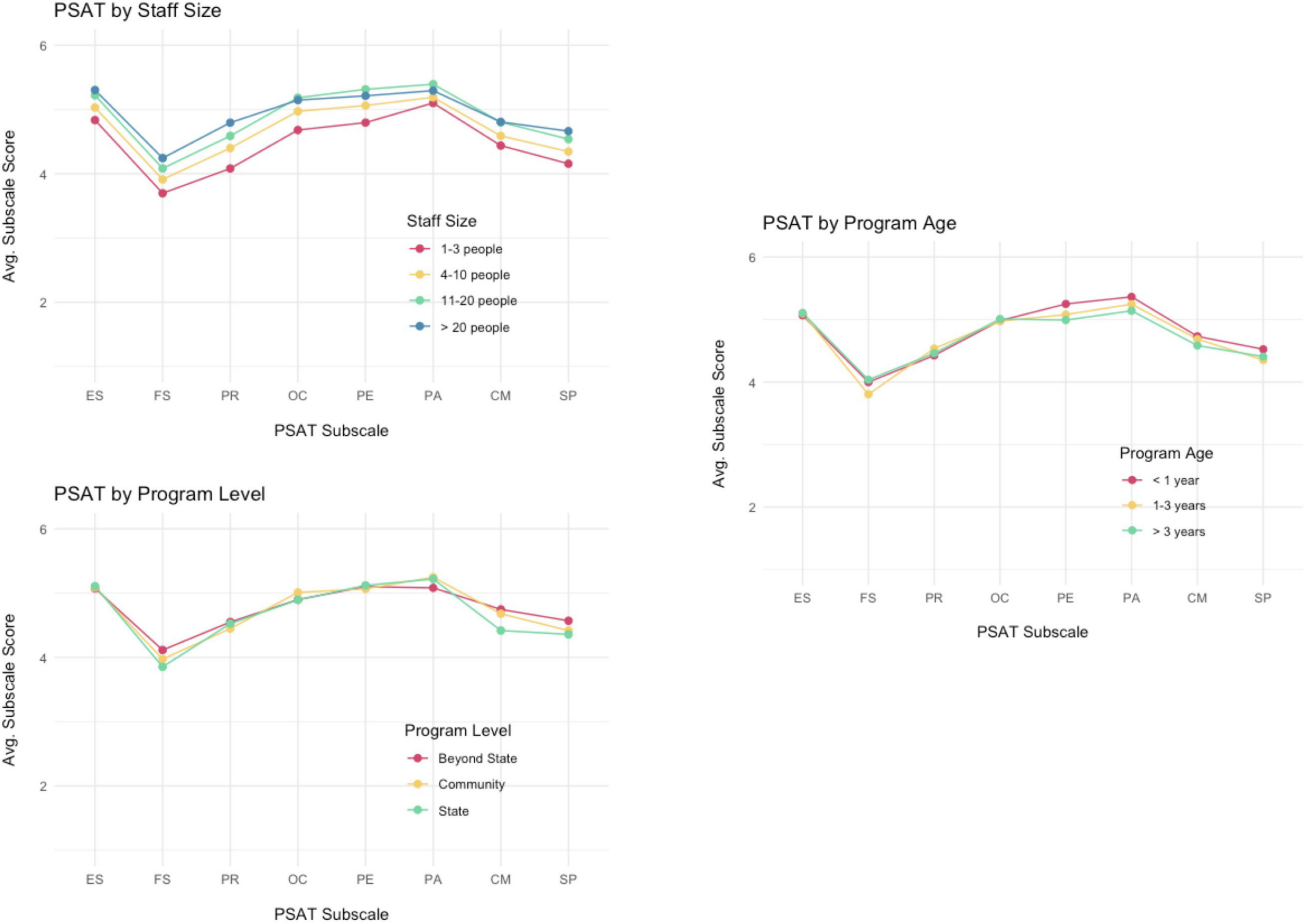
PSAT subscale scores by program level, staff size, and program age.

Additional analyses were conducted to understand how program size, age and level impact sustainability capacity (Table 3). Overall sustainability capacity was significantly related to program size (*F* = 25.6; *p* < 0.001). In general, larger programs (>20 staff and volunteers, *M* = 4.93) were perceived as more sustainable than smaller programs (< 4 staff and volunteers; *M* = 4.47). This pattern was apparent across all of the subscale domains as well-programs with three or fewer staff and volunteers reported significantly less capacity for sustainability compared to programs with 21 or more staff and volunteers for all eight of the subscales. Figure 4 shows the subscale means by size of the program, and there is a discernible dose-response pattern where larger staff sizes are associated with higher PSAT subscale scores.

In comparison, program level and program age were not as strongly or consistently related to sustainability capacity scores. Overall sustainability capacity was not associated with program age (*F* = 1.49; *p* = 0.226). However, older programs (>3 years, *M* = 4.04) reported higher capacity for funding stability (*M* = 4.04; *F* = 7.03; *p* = 0.001), while younger programs (< 1 year) showed greater capacity in program evaluation (*M* = 5.25; *F* = 8.54; *p* = 0.000) and program adaptation (*M* = 5.36; *F* = 7.26; *p* = 0.001).

Overall program sustainability capacity did not vary significantly by program level (*F* = 0.61; *p* = 0.54). However, state-level programs reported the lowest level of communications capacity (*M* = 4.42; *F* = 6.95; *p* = 0.001) while higher level (beyond state) programs reported higher financial stability (*M* = 4.11; *F* = 3.06, *p* = 0.047).

## Discussion

This study evaluated the PSAT continued performance in two major areas: first, through assessment of reliability and measurement structure, and second, in understanding some program characteristics that affect programs' sustainability capacity. The PSAT maintained its excellent reliability when tested with a larger and more diverse sample over time, further solidifying it as a reliable tool for assessing sustainability capacity. Initial criterion validity was explored through the assessment of program characteristics, including program type and program size. The data collected across differing programs and users reinforces the ability of the PSAT to assess sustainability capacity in relevant areas. The PSAT aids in assessment of many areas of public health, including those that address addiction and mental health programming.

The PSAT, therefore, remains a reliable and valid instrument for practitioners to use when assessing their program's sustainability capacity. While some work has adapted the PSAT for specific areas, this work suggests that the PSAT remains valid in its entirety, even when assessed in a larger and more diverse sample (31). While there have been other measures developed for sustainability capacity in certain areas or to assess sustainment, this capacity focused measure is both pragmatic and generalizable to different settings and amongst those in different roles, including both practitioners and research team members (5). This assessment responds to the need for reliable measures within implementation science, specifically in the area of sustainability research (1, 32, 33).

Further, this data provides information about real-world programs to support and enhance program sustainability. This allows for practitioners and researchers to better understand what constructs should be targeted to enhance program sustainability in public health, mental health, and clinical care.

Ultimately, this theoretically driven work helps move from considerations about definition into better understanding of measurement of this construct. Next, studies of sustainability need to focus more about prediction and mechanisms on which sustainability acts. This study helps tie important information about theory and frameworks to data around these contextual factors that can drive sustainability capacity. These PSAT domains could inform future qualitative studies to explore the concepts further and elucidate how they could contribute to interventions to increase future sustainment.

A strength of this study is the large number of participants, even though the sample was comprised of those who sought out the measure for use. The time span covered by the sample further allows for strengthening of outcomes related to reliability and validity. Finally, this sample represents many types of programs as well as locations of assessment, including programs both within the United States and internationally. International participants were from countries including: Australia, Canada, and the United Kingdom. However, we do not collect specific location of sites through our online survey at this time. Additionally, this assessment was conducted prior to a Spanish translation of the measure being available, so is limited to English speaking respondents. Future validation work can expand on the initial variables used in this sample to assess criterion validity as well as explore PSAT responses in multiple languages.

The PSAT provides a reliable tool for assessing a program's sustainability capacity. In addition, the PSAT has been found to be easy to use, requiring no or minimal training (34). As a result, practitioners, evaluators, and researchers can use the PSAT in their sustainability planning efforts with confidence. This study further supports the reliability, validity, and usefulness of this instrument. While other instruments have been developed for specific settings, this tool assists with implementation practice and evaluating a wide variety of programs. This study does not connect sustainability capacity to sustainment outcomes, due to a lack of information about the sustainment metrics of the programs. Future research ought to investigate the link between sustainability capacity and sustainment outcomes.

Additionally, clinical settings often have been identified as having unique processes and structures to those in public health programs (6). For example, clinical settings are often less reliant on finances than public health programs. To assess these settings, an adaptation of the PSAT was developed that focuses on clinical programs and practices. The Clinical Sustainability Assessment Tool has also been translated for use in other languages and has been demonstrated as reliable in both domestic and global settings (35).

In addition to future work focused on broader dissemination of this tool for practice, there are opportunities to explore how different organization contexts influence program sustainability. The contexts within which different programs, such as public health and educational programs, are delivered can vary widely, even within similar geographic regions. Therefore, further work should focus on understanding this varying context, including differences in program level, populations, and settings, and their relationship to overall sustainability capacity. While other work has adapted this tool for specific clinical contexts, the PSAT should continue to be utilized and tailored for other audiences.

## Data availability statement

The raw data supporting the conclusions of this article will be made available by the authors, without undue reservation.

## Author contributions

CB led the design of the study and drafting the manuscript. SM assisted with planning the study, reviewing the data, and drafting the manuscript. KP provided supervision of the study and assisted with the design of the study and drafting the manuscript. RH assisted with data collection, data preparation, and editing the manuscript. MH assisted with the design of the study and drafting the manuscript. SD assisted with data collection and study design. DL led the data analysis and assisted with planning the study and drafting of the manuscript. All authors reviewed and approved the final manuscript.

## Funding

This work was funded by the Centers for Disease Control and Prevention, Office on Smoking and Health, contract no. 75D301-20-R-68063.

## Conflict of interest

The authors declare that the research was conducted in the absence of any commercial or financial relationships that could be construed as a potential conflict of interest.

## Publisher's note

All claims expressed in this article are solely those of the authors and do not necessarily represent those of their affiliated organizations, or those of the publisher, the editors and the reviewers. Any product that may be evaluated in this article, or claim that may be made by its manufacturer, is not guaranteed or endorsed by the publisher.
